# Expression of Concern: Down-regulation of XIAP enhances the radiosensitivity of esophageal cancer cells *in vivo* and *in vitro*

**DOI:** 10.1042/BSR-20170711_EOC

**Published:** 2020-07-27

**Keywords:** Esophageal cancer, Gene silencing, Radiosensitivity, siRNA, Viability, XIAP

The Editorial Office has been made aware of potential issues surrounding the scientific validity of this paper, hence has issued an expression of concern to notify readers whilst the Editorial Office investigates.

